# The impact of race relations on NFL attendance: An econometric analysis

**DOI:** 10.1371/journal.pone.0226938

**Published:** 2020-01-24

**Authors:** Nicholas Masafumi Watanabe, George B. Cunningham

**Affiliations:** 1 University of South Carolina, Columbia, South Carolina, United States of America; 2 Texas A&M University, TX, United States of America; Valparaiso University, UNITED STATES

## Abstract

Recent protests by athletes focused on raising awareness of social issues and injustices, such as the Black Lives Matter protests led by Colin Kaepernick of the National Football League’s San Francisco 49ers, have generated a great deal of attention and debate within society. Notably, the protests conducted by these players before games in the 2016 and 2017 seasons became such a sensational topic, that extraordinary amounts of attention was paid to it by the media, consumers, and even politicians who often denounced the players as being unpatriotic. Against this backdrop, the current research examines whether fluctuations in attendance at National Football League games are associated with explicit attitudes towards race, implicit racial prejudice, and racial animus within a population. Specifically, using multiple measures of racial attitudes as part of an econometric model estimating attendance at games, the results suggest that having a higher level of implicit bias in a market leads to a decline in consumer interest in attending games. Additionally, using interaction effects, it is found that while protests generally reduced the negative effects of implicit bias on attendance, markets with lower levels of implicit bias actually had greater declines of attendance during the protests. From this, the current study advances the understanding of racial attitudes and racial animus, and its impact on consumer behavior at the regional level. That is, this research highlights that racial sentiments in a local market were able to predict changes in market behaviors, suggesting that race relations can have wide reaching impacts.

## Introduction

In 2016, San Francisco 49ers quarterback Colin Kaepernick sat during the pre-game playing of the “Star-Spangled Banner”—a time other National Football League (NFL) players, coaches, and fans have traditionally stood. In later games during the season, he took a knee during the playing of the anthem. Explaining his actions, Kaepernick commented: “I am not going to stand up to show pride in a flag for a country that oppresses black people and people of color…. To me, this is bigger than football and it would be selfish on my part to look the other way. There are bodies in the street and people getting paid leave and getting away with murder” [1]. Though Kaepernick initiated the protest, he was not alone in engaging in such actions. Approximately 200 players peacefully protested prior to the games in the third week of the 2017 season [2]. Players and coaches in other leagues, including LeBron James of the National Basketball Association (NBA), San Antonio Spurs (NBA) head coach Greg Popovich, and the entire Minnesota Lynx team (Women’s National Basketball Association), were among those who used their platform as athletes to engage in peaceful protest [3].

Though protests spread around several sport sites, those in the NFL arguably generated the most discussion, debate, and action. The protests even drew attention from President Trump, for example, who noted in a speech in Alabama that he would like to see NFL owners terminate the employment for those who disrespected the flag [4]. Many national news agencies conducted polls of the American public, showing that nearly half of the respondents disapproved of the NFL protests [4]. Alongside the dissension was a decrease in attendance. A Rasmussen study conducted during the 2016 season indicated that nearly a third of Americans were less likely to watch NFL games following the protests [5]. The behavioral intentions of the respondents were seemingly reliable predictors of their future behaviors: from the 2016 to the 2018 season, average gameday attendance dropped 3.6 percent, or roughly 2400 fans per contest [6]. A drop in attendance was not the only outcome. The 2016 season was the final one Kaepernick played, despite having the fifth best touchdown-to-interception ratio of all time [7].

What, though, precipitated the attendance decline? Did protests during the national anthem spark feelings of patriotism? President Trump suggested as much, noting that his opposition was based on his love of country, and he tweeted: “respect for our Country, Flag and National Anthem. NFL must respect this!” (as cited in [8]). [4], in her analysis of the national polls, further showed that, although the protesting players cited the desire to raise awareness of police violence and systemic racism, Americans largely reported seeing the protests as disrespecting the US flag and US military. Further analysis of the data, however, showed deep racial divides in how people responded [4]. Black Americans approved of the protests more so than Whites, and the differences were by a margin of over two-to-one in some polls. A similar poll by the *Texas Tribune* showed that almost half of the respondents had an unfavorable view of the NFL. However, Whites were twice as likely as Blacks to express such antipathy [9]. These differences parallel related polling from the Pew Research Center showing that Blacks are 55 percent more likely than Whites to see racism as a “big problem” in America [10].

These data suggest that race relations and racism might contribute to the drop in NFL attendance, especially following the protests. The purpose of this study was to examine such a possibility. In drawing from theories related to explicit attitudes towards race and implicit racism [11, 12, 13], we examine the role of contemporary forms of racism in predicting NFL attendance. In doing so, we consider implicit racial bias [14] and racial animus [15], and show that implicit bias reliably predicts fluctuations in game attendance, even when controlling for other traditional predictors of sport demand. In the following sections, we offer our theoretical foundation and present specific hypotheses.

## Theoretical framework

### Forms of racial prejudice

#### Explicit bias

In this study, we focus on racial prejudice and its various manifestations. We begin with a discussion of explicit racial prejudice. Historically, people have considered racial prejudice as a negative attitude explicitly directed toward an out-group. For example, [16] wrote that prejudice is “antipathy based on faulty and inflexible generalization” (p. 9). More recently, [17] wrote that prejudice reflected “a negative evaluation of a group or an individual on the basis of group membership” (p. 359). People can consciously and deliberately maintain these negative attitudes, or what social psychologists refer to as explicit forms of racism [12, 13]. For example, Gallup, a polling agency in the US, has for decades asked participants about their attitudes concerning various racial issues. When people respond to questions of race or racial equality, they are drawing on their explicit attitudes (for an overview of their polls, see [18]).

Though polling agencies and researchers commonly ask people about their explicit racial attitudes, there are potential flaws to this approach. First, since the mid 20^th^ century, people have been hesitant to express explicit attitudes because of social norms against doing so [19]. Indeed, [17] have shown that explicit racial attitudes are closely linked with societal approval of such expressions. These dynamics explain, for instance, why people might freely convey their negative attitudes toward individuals who harm children but condemn prejudice based on demographic characteristics. Second, and related to the first point, one’s failure to admit racist attitudes does not mean the individual does not hold them. To be sure, there are times when the lack of reported racism truly reflects the absence of racist beliefs; in other cases, though, people might feel motivated to suppress their prejudice or deny its existence, even though the prejudice remains [20]. Finally, it is possible that people believe they do not hold racist beliefs but actually do at an unconscious level. In this case, they are likely to report a lack of racist attitudes but nevertheless express prejudice when certain stimuli are present [21]. In each of these cases, prejudice is underreported, and the link between explicit attitudes and subsequent outcomes is attenuated.

### Racial animus

Noting these shortcomings, a number of authors have explored other forms of racism, the first of which is racial animus. We consider racial animus as explicit racial prejudice that people might not otherwise share with others or admit to harboring. Like other forms of explicit prejudice, people consciously maintain racial animus; however, unique to this form of bias is the desire by the individual to hide such attitudes or express them in private.

Consistent with our previous arguments, [15] suggested that people are frequently unwilling to admit they have socially unacceptable attitudes (e.g., racist attitudes) to survey takers; thus, large social surveys likely under-estimate the number of racists and underestimate racism’s effects. Seeking to rectify this shortcoming, he argued for a different approach—assessing people’s searches on Internet search engines, such as Google. Consider, for example, that people use their computers or devices in relative privacy, and consequently, they might search for material—from pornographic material, to ways to harm themselves, to racist jokes, among others—that they would not otherwise share with another person [22, 23, 24]. If this is the case, then Internet searches actually offer a truer estimation of one’s attitudes than do surveys [25].

Drawing from this possibility, [15] examined Internet searches to develop a measure of racial animus. Using the Google Trends tool, he considered racial animus as the percent of Google searches that included racial slurs (the N-word). He found that the new measure of racial animus successfully predicted votes for Barack Obama in the US presidential elections. Stephens-Davidowitz’s racial animus measure was also a stronger and more reliable predictor than were self-reported racist attitudes, as measured through social surveys. Other scholars have observed similar patterns, such that Google searches for the N-word are associated with all-cause Black mortality rates [26], as well as pre-term births and low birthrates among Black mothers [27].

### Implicit bias

Finally, in addition to racial animus, racial prejudice might take the form of implicit attitudes. Implicit attitudes differ from more explicit forms of racial prejudice, whether explicit racial prejudice or racial animus, in several ways. Implicit attitudes are automatic responses to stimuli [11], whereas more explicit forms of prejudice are consciously and deliberately maintained. Implicit racial prejudice manifests when there is a match between an external stimulus and the individual’s association set linking the stimulus with particular characteristics. Thus, unlike with explicit forms of racial prejudice, people who express implicit racial prejudice believe they are not racist and support egalitarian values; nevertheless, they hold unconscious negative feelings and beliefs toward racial minorities. From this, some scholars have argued that patterns of discriminatory behavior towards other races may be linked to implicit racial prejudice [28], which is more prevalent than explicit racism in the population [14].

Not surprisingly, implicit and explicit attitudes are generally not related to one another. For example, in a meta-analysis focusing on ethnic and racial prejudice, [29] found that explicit and implicit measures of racial prejudice were weakly correlated (.14). Finally, in a recent series of commentaries, [30, 31] persuasively argued that implicit attitudes take on group level properties and can predict behavioral outcomes at the group level—these are arguments not necessarily reflective of more explicit forms of prejudice. At the same time, other researchers have argued that implicit bias does not necessarily have a stronger impact on race-based attitudes and behaviors [32]. Notably, in a meta-analysis of the research conducted on both implicit and explicit measures of racial bias, it was found that scores of implicit bias tended to perform worse than explicit measures [29]. Overall, there is clearly a divide within the literature, as scholars examining the same context continue to find conflicting evidence in regards to whether implicit or explicit measures of racial bias have a more powerful effect on the behaviors of individuals and society as a whole [33, 34].

### Racial attitudes and NFL attendance

We suspect that, broadly speaking, people’s racial prejudice is associated with NFL attendance following the Black Lives Matter-related protests. Our rationale is based largely on the race of the participants and the focus of the protests. Though there were exceptions (e.g., Chris Long of the Philadelphia Eagles), most of the NFL protesters were Black, and they were protesting the killing of Black individuals in the US. Researchers have found that Whites and Blacks widely differ in their support of Black Lives Matter, in general, as well as the degree to which they believe Black Lives Matter initiatives will ultimately be effective [35]. Given this racial divide, it is possible that the protests spurred racist attitudes among NFL consumers, thereby resulting in decreased attendance.

The foregoing discussion of racial prejudice suggests, however, that there are likely differences in the predictive validity of different racism measures. With respect to racial animus, recall that few people are likely to admit they are racist, even if they harbor such attitudes [19]. In the context of the current study, this perspective would suggest that people are unlikely to admit that their opposition to the protests, or their decision to forego game attendance, is racially motivated. As a result, using social science surveys in predicting subsequent race-related outcomes, such as support for Black political candidates [25] or, in this case, NFL football, might not be efficacious. On the other hand, people are more likely to express their racial prejudice in private, such as through their Internet searches [25]. Instead, as we have previously argued and [15] has shown in the context of voting patterns, their Internet searchers likely represent a truer picture of their racial attitudes. Given this connection, we formulate the following research question:

**Research Question 1**: Does a newly-created measure of racial animus predict attendance?

We also expected that implicit racial prejudice would predict NFL attendance. [36], in their meta-analysis, showed that implicit racial prejudice, as measured by the implicit association test (IAT), explained behavioral outcomes beyond explicit measures of prejudice. Several years later, [29], in their meta-analysis, offered evidence to the contrary, finding that neither explicit or implicit racial prejudice were associated with racial discrimination. In both cases, the magnitude of association was small.

[30, 31], in their recent analyses, offer evidence why implicit racial prejudice might be associated with subsequent race-related behaviors, particularly at the group level. They noted that most analyses focusing on racial prejudice and discrimination take place at the individual level, and the associations are modest. The meta-analyses we just reviewed confirmed this position. On the other hand, when the analysis takes place at the group, metropolitan, state, or national level, implicit bias is strongly associated with subsequent outcomes. They point to context to explain these findings: “we believe it is more accurate to consider implicit bias as a social phenomenon that passes through the minds of individuals but exists with greater stability in the situations they inhabit” [30], p. 236). In this way, implicit bias is a social phenomenon that takes on shared properties of the larger group, based on the specific situation and context. Cultural and a shared understanding of commonly held stereotypes contribute to this end [37]. [30] argued, “the near-universal knowledge of stereotypes creates the potential for anyone to experience implicit bias” (p. 237).

Because the implicit attitudes are widely shared, they are likely to be predictive of outcomes measured at the group level. Indeed, researchers have shown that metropolitan regions with high levels of implicit racial attitudes also saw racial disparities in police shootings within that area [38]. In another study focusing on implicit attitudes, [39] found a different pattern of implicit prejudicial attitudes related to weight, depending on the level of analysis. Specifically, overweight individuals had low levels of implicit weight bias, but at the country level, implicit bias was positively associated with the populace who are overweight.

These findings are relevant to our analysis, which takes place at the metropolitan level of analysis. Drawing from [30, 31], and the recent work on group-level implicit prejudicial attitudes and subsequent negative behaviors toward out-group members, we suspected that as implicit prejudice in a metropolitan area increased, attendance at NFL games following the Black Lives Matter protests would decrease. As such we develop the second research question:

**Research Question 2**: Do IAT scores predict attendance?

Finally, as prior studies have argued that measures of explicit racial bias [32, 33] can have a stronger impact on the behaviors of individuals within a population, we develop a third research question in line with the previous two:

**Research Question 3**: Do explicit measures of belief about race-relations predict attendance?

## Method

In order to examine the research questions, we develop a model to estimate attendance demand guided by prior theoretical and empirical studies [40, 41]. To begin with, empirical studies analyzing the demand for professional sport have typically used either reported attendance [40, 42], the natural log of attendance [43, 44], or the percent of stadium capacity [45, 46] as the dependent variable in their models. In order to develop these dependent variables, attendance and capacity data was collected for every regular season game from the 2012 through 2017 NFL seasons. Because this analysis focuses on the impact of racial animus and bias on attendance, the sample is constrained to domestic games played within the United States. As such, 18 international games were removed from the dataset, as well as all home games played by the Chargers, as their relocation to a soccer stadium as they awaited the completion of their new stadium severely constrained demand. From this, the final data set for this research is composed of 1,472 observations of home attendance for regular season games over a six-year period.

Turning focus to the independent variables in our model, we first create a measure of racial animus for each market with an NFL franchise by following methods pioneered [15]. Specifically, using the Google Trends website, we perform searches for the singular and plural form of the “N-word” for each market [15]. However, because Google Trends normalizes all search data, [15] and other scholars [47] have addressed this issue using common search terms, such as “weather.” Specifically, three searchers are conducted for a market using the “N-word”, the common search term of “weather” and then a combined search for both the “N-word” and “weather.” From this, the values are then adjusted using the equation:

RacialAnimus=NWord−(Weather−(NWord+Weather)) (1)

Following this, researchers then use an estimation method to weight these values when there is missing data because of the lack of searches in smaller markets. However, for this research, as the markets being used in this study are all cities with relatively large populations, there were no values of 0 observed in the data. As such, there is no need for further adjustments or weighting using the regression methods laid out [48]. In this way, the formula from Eq 1 is used to develop an adjusted score for each market that takes into account the relative volume of searches for the “N-word.” From this, it provides us with the variable *RacialAnimus*, measuring how often individuals in a local market search for the “N-word” in the week before a game.

As a measure of explicit attitudes towards race, we construct the variable *PewPct*. Particularly, this measure is created using data from recent Pew Research Center studies on race relations in America. Specifically, *PewPct* signifies the percentage of individuals in each state who stated that race relations were becoming worse in the U.S. It should be noted that *PewPct* does have the limitation of not explicitly asking respondents about prejudice, and thus does not provide complete certainty in regards to measuring whether an individual displays explicit prejudice. That is, it is possible for respondents to not be prejudiced against others, but still have the feeling that race relations are regressing in the country. As such, *PewPct* measures the general perceptions of the public about race relations, rather than measuring the actual percentage of individual who exhibit explicit prejudice.

Moving along to the variable accounting for implicit racial bias, we utilized data from the racial Implicit Association Test (IAT), an empirical methodology developed by psychologists to measure bias [49]. Notably, prior IAT research has shown that small effects can have larger societal impact, especially in regards to race [50]. Based on this, we develop the variable *IAT* from yearly racial IAT tests conducted by Harvard University and Project Implicit from 2002 to 2017. Specifically, we utilize the data from 2012 through 2017 to calculate a yearly racial IAT score for each state that has an NFL team, with higher values of *IAT* indicating greater implicit bias in the population. As such, the inclusion of this variable allows for examination of whether there is a statistical relationship between the level of implicit bias exhibited by individuals in the local population and attendance at NFL games. Furthermore, it allows us to control for levels of implicit bias when examining the effect that racial animus and explicit attitudes have on attendance. In this manner, the final model includes three different measures of attitudes towards race.

Continuing to the other independent variables within this research, prior studies of attendance demand in sport highlight the need to control for the performance of teams, local market potential, as well as other structural influences [51, 52]. As such, we develop a number of variables to account for these potential determinants of demand [40]. First, in considering the performance of teams, studies of attendance in the NFL and other sport leagues have used a variety of metrics to capture the quality of contests, including the percentage of games won [53], the number of points scored by the home team [54], as well as the home team record [55]. As the quality of the home and away team may play a role in determining fan interest in attending games [56], we utilize the lagged win percent for both the home (HomeWPCTLag) and away (AwayWPCTLag) teams. The use of a lagged variable accounts for the fact that fans are not aware of the outcome of a game when they purchase tickets, and thus the decision to attend games are likely driven by prior team performance.

Next, to control for the market characteristics, we utilize data from the Bureau of Economic Analysis (BEA) to build measures of population (*Population*) and per capita income (*PCI*) for each market NFL market. Because this study considers race relations, in addition to the inclusion of above market metrics commonly used in econometric modeling [57], we develop two additional variables that account for other demographic factors. As the racial demographics of a market may influence the level of bias in that region [58], we control for the percentage of minorities (*MinorityPct*) within each Metropolitan Statistical Area (MSA) using Census data estimates from the American Fact Finder website. Finally, as the voting patterns from residents in a market can influence attendance after protests [44], we measure the percentage of individuals in each MSA who voted for Republican candidates (*GOPPct*) in the most recent Presidential election.

The last group of variables included in the model are those that account for various structural factors that could influence attendance at sporting events [51]. To control for the timing of games, we include dummy variables to measure if teams played games on a weekday (*Weekday*) or a holiday (*Holiday*). Additionally, as prior research has found the presence of rivals can increase fan interest in attending games, the *Division* variable was developed to measure whether games were played against divisional opponents who are viewed as rivals. Finally, considering that the novelty of having a team relocate to a new market can increase attendance [59], we use a dummy variable to account for the home games of teams that moved to a new location (*Relocated*). Similarly, because the construction of new stadiums can also have a novelty effect, we measure the age of the stadium hosting a game (*StadiumAge*), as well as included the square of stadium age (*StadiumAgeSq*) to consider whether the effect of age changes over time. These two stadiums variables are commonly used in empirical models estimating attendance in professional sport leagues, as it has been found that there is a concave relationship between stadium age and attendance [60, 56]. That is, sport fans have been shown to have a preference for new stadiums in their first few years of operations, or older classical stadiums, typically at least five to six decades in age or older [61].

Finally, to account for the protests that occurred in the NFL, we include the variable *Protest*, which simply measures as 1 in years that there were mass protests in the NFL (2016 and 2017), and a 0 for the years before the protests (2012–2015). Additionally, in order to consider the three hypotheses examining whether racial animus and implicit racial bias affects NFL attendance, we develop interaction variables by multiplying the racial measures with *Protest*, thus estimating whether the protests had any effect on the racial measures on attendance. Thus, the inclusion of the interactions allows for the analysis of whether racial animus or implicit bias have a significant relationship with attendance, and whether these patterns change based on events in the league. From the inclusion of these interaction variables, it then allows us to formulate the following research questions:

**Research Question 4:** Does racial animus interact with the occurrence of protests to predict attendance?
**Research Question 5:** Do IAT scores interact with the occurrence of protests to predict attendance?
**Research Question 6:** Does explicit racial attitudes interact with the occurrence of protests to predict attendance?

In this manner, as our dataset covers the four seasons before the Black Lives Matter protests (2012 through 2015) and two seasons in which players protested (2016 and 2017), thus providing the ability to examine whether explicit attitudes towards race or implicit bias influenced attendance at NFL games before or after the protests. As such, through including the above variables, the general model for NFL game attendance in this study takes the form of:

$$\begin{array}{l} {Attendance}_{it}=\alpha_{0}+\beta_{1}{RacialAnimus}_{it}+{\beta_{2}{PewPct}_{it}+\beta_{3}{IAT}_{it}+\beta }_{4}{HomeWPCTLag}_{it}+\beta_{5}{AwayWPCTLag}_{it}+\\ \beta_{6}{Population}_{ijt}+\beta_{7}{PCI}_{it}+\beta_{8}{MinorityPct}_{it}+\beta_{9}{GOPPct}_{it}+\beta_{10}{Weekday}_{it}+\beta_{11}{Holiday}_{it}+\beta_{12}{Division}_{it}+\\ \beta_{13}{Relocated}_{it}+\beta_{14}{StadiumAge}_{it}+\beta_{15}{StadiumAgeSq}_{it}+\beta_{16}{Protest}_{it}+\beta_{17}{RacialAnimus\;x\;Protest}_{it}+\\ \beta_{18}{PewPct\;x\;Protest}_{it}+\beta_{19}{IAT\;x\;Protest}_{it}+\mu_{it} \end{array}$$ (2)

where i indicates NFL teams and t indicates the season. The summary statistics for the variables included in the model can be found in Table 1.

**Table 1:** Summary statistics.

	Full Data Set			Pre-Protests			During Protests		
Variable	Observations	Mean	Std. Dev.	Observations	Mean	Std. Dev.	Observations	Mean	Std. Dev.
Attendance	1,472	68,579	8,279	983	68,290	8,397	489	69,162	8,014
AttendancePct	1,472	0.9688	0.0722	983	0.9654	0.0741	489	0.9754	0.0678
lnAttend	1,472	11.12838	0.1221	983	11.12379	0.1254	489	11.13762	0.1146
Population	1,472	4,915,659	4,601,295	983	4,757,108	4,515,651	489	5,234,382	4,757,532
Per Capita Income	1,472	52,110	9,459	983	51,164	9,185	489	54,011	9,723
MinorityPct	1,472	0.3058	0.1038	983	0.3025	0.1031	489	0.3125	0.1051
GOPPct	1,472	0.4224	0.0983	983	0.4245	0.0974	489	0.4183	0.1000
WPCTLagHome	1,472	0.4854	0.2679	983	0.4704	0.2731	489	0.5156	0.2545
WPCTLagAway	1,472	0.5014	0.2653	983	0.4910	0.2700	489	0.5222	0.2547
Weekday	1,472	0.1359	0.3428	983	0.1343	0.3411	489	0.1391	0.3464
Holiday	1,472	0.0272	0.1626	983	0.0153	0.1226	489	0.0511	0.2205
Division	1,472	0.3784	0.4852	983	0.3784	0.4852	489	0.3783	0.4855
Relocated	1,472	0.0048	0.0688	983	0	0	489	0.0143	0.1189
StadiumAge	1,472	21.74	19.70	983	21.04	18.36	489	23.15	22.11
StadiumAgeSq	1,472	865	1,753	983	779	1,533	489	1,038	2,120
Protest	1,472	0.3322	0.4712	983	0	0	489	1	0
RacialAnimus	1,472	38.51	13.59	983	37.89	13.21	489	39.76	14.25
PewPct	1,472	0.4845	0.0395	983	0.4889	0.0424	489	0.4757	0.0313
IAT	1,472	0.3018	0.0309	983	0.3096	0.0296	489	0.2861	0.0275
Capacity	1,472	70,615	6,656	983	70,427	6,267	489	70,993	7,370

Before estimating the results from the model in Eq 2, there is first need to consider the method of estimation and other potential econometric issues. To begin with, focusing on the dependent variable of *Attendance*, plotting the data in a histogram reveals that the data are not normally distributed, and thus could produce biased results when estimating a regression [44]. To account for this, we follow the approach commonly utilized in economic research on sport demand by transforming *Attendance* by its natural log to estimate the regression results [56]. Because there are a large number of sellouts in the NFL, attendance numbers are constrained by the size of the stadium in which a team plays. When there are a significant number of sellouts in a sport league, it can result in biased estimates from a regression because it does not account for the potential that teams could have had greater demand if they were not constrained in terms of stadium capacity [62]. For example, in their examination of football attendance in the U.S., [45] utilized a Tobit regression because 25 percent of the games in their sample were sell outs, thus resulting in the need to censor right-hand side observations. For the sample within this study, home teams on average filled over 97 percent of their stadium capacity, thereby suggesting that use of an Ordinary Least Squares (OLS) regression is not a suitable method of estimation. The common approach within the academic literature to address this issue is using a Tobit regression with the upper limit set at stadium capacity for each game, as this censors observations of sold out games [45, 63, 64]. Furthermore, as the Breusch-Pagan test was statistically significant (*p* < 0.01), there is need to account for the presence of heteroscedasticity [65]. We adjust for this using homoscedastic consistent standard errors clustered by team, which also accounts for any correlations in the standard errors for individual teams over time [66, 67]. Based on the above, we estimated the results of the Tobit regression using the STATA 15 statistical software. Notably, five different models were estimated, starting with a full model including all variables, a reduced form model with no interaction terms, and then three separate regressions utilizing only one of the racial measures as well as its interaction term. Additionally, we utilized several estimation strategies, including the use of fixed or random effects, and various transformations of the dependent variable to provide as complete a picture as possible in examining whether racial animus and implicit bias have an impact on attendance at NFL games. Overall, the results held mostly consistent between the models, and thus we focus on findings from the Tobit regressions following the guidance of prior sports economics research.

## Results

Turning focus to the first research question examining the relationship between racial animus and attendance at NFL games, the results from the estimated model (Table 2) find that *RacialAnimus* was insignificant in relation to attendance. These results suggest that there was no relationship between attendance at NFL games and racial animus as measured by Google searches. Continuing to the second research question focused on whether levels of implicit racial bias will cause a decline in attendance at NFL games, the *IAT* variable was found to be negative and significant in all models. In considering the coefficients for IAT, which can be understood in terms of percent change in attendance, the results ranged from -0.81 to -0.98, suggesting that a one point change in the IAT scores for a market caused a decline in attendance of about 81 to 98 percent. As the variation in IAT scores in this study tends to be relatively small, with the greatest observed variance from the mean being about 0.1, it would indicate that this would lead to a change in NFL attendance of about 8.1 to 9.8 percent in some markets. Overall, these findings provide evidence that *IAT* scores of a market had a relationship with attendance, indicating that when there was greater implicit racial bias in a market, individuals were less likely to attend games.

**Table 2:** Tobit regression results (DV = lnAttendance).

	Model 1		Model 2		Model 3		Model 4		Model 5	
Variables	Coeff.	Std. Err.	Coeff.	Std. Err.	Coeff.	Std. Err.	Coeff.	Std. Err.	Coeff.	Std. Err.
Population (x 1,000,000)	0.0012	0.0049	-0.0003	0.0048	-0.0034	0.0047	-0.0041	0.0047	0.0019	0.0049
Per Capita Income (x 1,000)	-0.0047***	0.0015	-0.0040**	0.0014	0.0003	0.0011	0.0007	0.0011	-0.0050***	0.0014
MinorityPct	0.4428***	0.1329	0.4243***	0.1312	0.3581***	0.1308	0.3929***	0.1313	0.4003***	0.1313
GOPPct	-0.3924**	0.1641	-0.2701*	0.1555	-0.1972	0.1550	-0.1212	0.1526	-0.4036**	0.1597
WPCTLagHome	0.0321***	0.0078	0.0305***	0.0077	0.0366***	0.0078	0.0335***	0.0078	0.0363***	0.0077
WPCTLagAway	0.0242***	0.0068	0.0240***	0.0068	0.0235***	0.0069	0.0241***	0.0069	0.0236***	0.0068
Weekday	0.0105*	0.0056	0.0114**	0.0056	0.0125**	0.0057	0.0120**	0.0057	0.0114**	0.0056
Holiday	0.0085	0.0131	0.0050	0.0127	0.0080	0.0132	0.0102	0.0127	0.0077	0.0126
Division	0.0035	0.0037	0.0035	0.0037	0.0032	0.0038	0.0035	0.0038	0.0035	0.0037
Relocated	0.3052***	0.0311	0.3008***	0.0311	0.3131***	0.0315	0.3135***	0.0314	0.3053***	0.0311
StadiumAge	-0.0061***	0.0008	-0.0063***	0.0008	-0.0055***	0.0008	-0.0059***	0.0008	-0.0058***	0.0008
StadiumAgeSq (x 1,000)	0.0465***	0.0113	0.0519***	0.011	0.0513***	0.0107	0.0564***	0.0109	0.0434***	0.0111
Protest	-0.0617	0.0761	0.0038	0.0050	0.0068	0.0123	0.0000	0.0708	-0.0992**	0.0427
RacialAnimus	0.0001	0.0002	0.0001	0.0002	0.0002	0.0002	---	---	---	---
RacialAnimus x Protest	-0.0001	0.0003	---	---	0.0000	0.0003	---	---	---	---
PewPct	0.2328***	0.0696	0.1945***	0.0610	---	---	0.1866***	0.0685	---	---
PewPct x Protest	-0.1089	0.1530	---	---	---	---	0.0189	0.1469	---	---
IAT	-0.9318***	0.1469	-0.8151***	0.1400	---	---	---	---	-0.8917***	0.1451
IAT x Protest	0.4265***	0.1531	---	---	---	---	---	---	0.3529**	0.1466
constant	11.65***	0.1448	11.56***	0.1389	11.17***	0.9629	11.03***	0.1076	11.78***	0.1388

*—significant at the 10% level

**—significant at the 5% level

***—significant at the 1% level

Next, considering the third research question asking whether explicit measures of belief about race relations can predict attendance, the *PewPct* variable was positive and significant in all models in which it was included (Table 2). Examining the results from the Tobit models, the positive coefficient for *PewPct* suggests that the higher the percentage of people in a market who felt there was race relations in the country were negative, the greater the attendance at NFL games. In this manner, the findings suggest that markets that had worsening attitudes towards race relations in the country, actually lead to an increase in consumer interest in purchasing tickets to attend NFL games. However, as previously noted, because the measure of *PewPct* does not directly measure the number of people who display explicit prejudice, the findings do not confirm that explicit bias increases consumer demand for sport.

Moving to the fourth research question focused on whether the interaction between the protests and racial animus was able to predict attendance, it was found that this interaction was insignificant in all models in which it was included. In this manner, the regression results indicate that the occurrence of the protests did not have any impact on the effect of racial animus on attendance.

**Fig 1:**
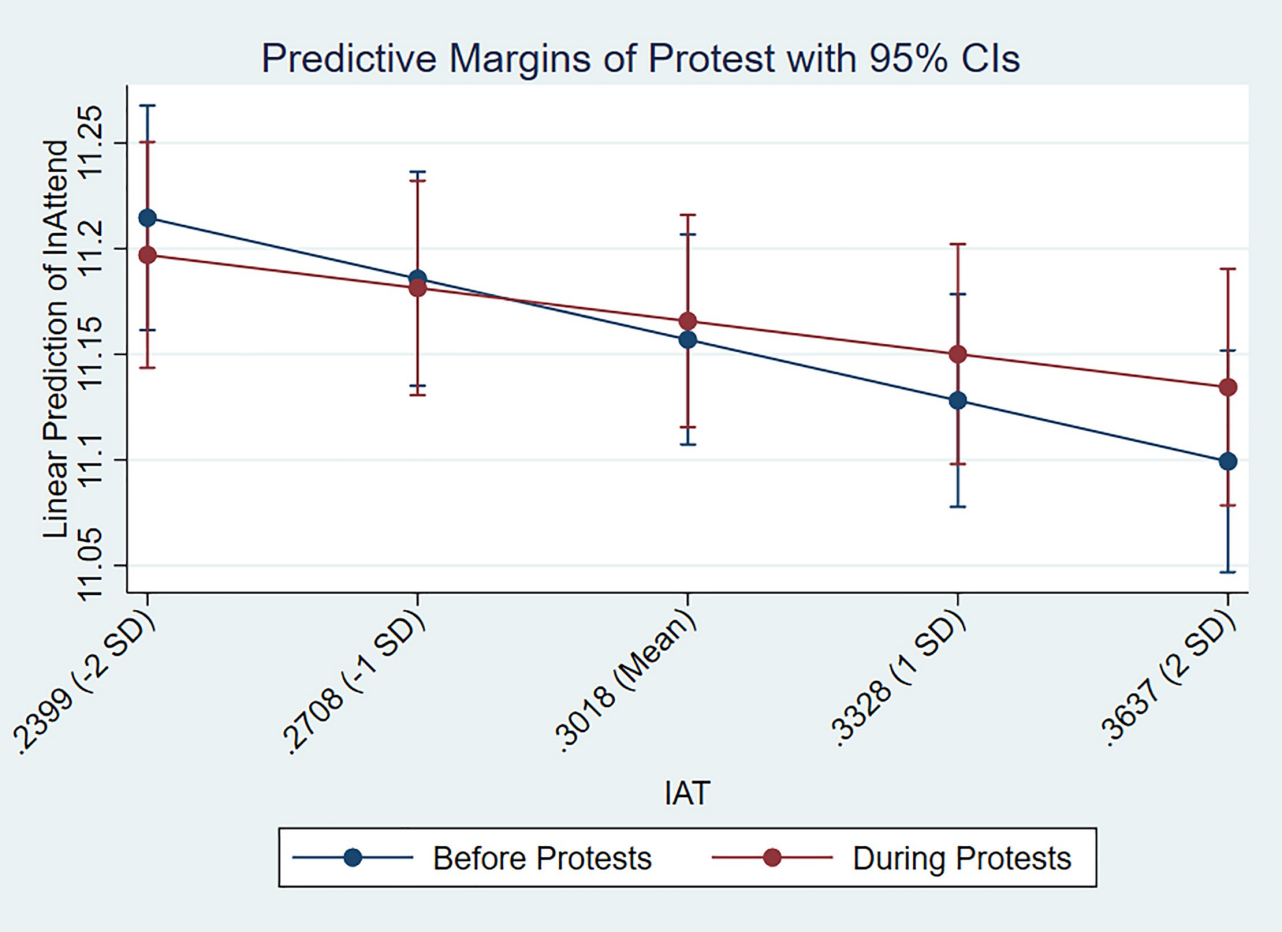
Predictive margins before and during the protests.

Next, for the fifth research question considering the interaction between IAT scores and the protests, the interaction was positive and significant. This finding suggests that during the protests markets with higher IAT scores experienced an increase in attendance compared to periods when there was no protests. To better understand this interaction, we utilized marginal effects (sometimes called partial effects), a method to measure “the effect on the conditional mean of y of a change in one of the regressors ([68], p. 333). The use of marginal effects is beneficial in this context because it provides a more intuitive manner through which to examine the results for interaction terms that include categorical variables [69]. That is, it allows for graphical representation of the difference in slopes between various groups, as well as the calculation of simple slopes to probe the results for interaction variables. Based on this, we calculate the partial derivatives for the interaction of IAT and protests, to provide us with the marginal effects. Specifically, we calculate the marginal effect at five points: first the mean value of IAT (0.3018), then at one standard deviation (0.2709 & 0.3328) from the mean, and finally at the second standard deviation from the average IAT score (0.2399 & 0.3637). From this, we then plot out the trend line for the marginal effects for before and during the protests, which are displayed in Fig 1.

In addition to plotting the two trend lines, we also calculated their simple slopes in order to probe the results of the interaction term. The slope for IAT before the protests (blue line) was -0.932 (*p* < 0.01) which closely matches the coefficient of the IAT variable within the first Tobit model. Next, the slope for IAT during the protests (red line) was -0.505 (*p* < 0.01). Although, this value does not initially seem to match the coefficient for the interaction in Model 1, the positive coefficient for the interaction of IAT and protests should be understood as the difference between the slopes for IAT before and during the protests. Thus, by subtracting the slope of IAT before the protests from the slope of IAT during the protests, we find a similar value to the interaction coefficient (-0.505 - (-0.932) = 0.427). In further considering the interaction from a graphical perspective, because of the difference in intercepts between the two trend lines, Fig 1 suggests a more complex relationship than simply stating that protests diminished the negative effects of IAT during the protests. Notably, Fig 1 highlights that for markets approximately one standard deviation below the mean IAT score, attendance was higher before the protests. However, moving to IAT values higher than this point switches these findings, with the presence of protests having a greater predictive effect on attendance.

In further considering the interaction between IAT and protests, the coefficient was 0.4265, similar to the slope of 0.427 calculated from the marginal effects, indicating attendance would increase by about 42% when there was a one unit increase in the interaction value. Taking this value in consideration alongside the coefficients for just IAT, the greatest observed variation from the mean in the dataset was about 0.1, meaning that those markets with the highest IAT during the protests would have an increase in attendance of about 4.27 percent. However, as markets with IAT at this level would be expected to have a decline in attendance by about eight to nine percent, the presence of the protests would essentially cut the effect of IAT on attendance in half. Thus, the results suggest that while having higher implicit bias in a market reduced attendance at NFL games, this impact was lessened during the time when protests were occurring in the league. As such, it highlights that rather than causing a negative impact on demand for NFL games, the protests might have actually mitigated some of the negative effects from implicit bias.

Finally, for the sixth research question analyzing the interaction of explicit racial attitudes and attendance, the interaction between protests and *PewPct* was insignificant in all models. In this manner, it designates that the positive effect of explicit attitudes towards race on attendance was not affected by the presence of the protests. As such, the overall results provide little evidence in regards to racial animus having an impact on attendance at NFL games, including the time period during which players were conducting protests. At the same time, the results indicate that IAT may have had an effect on attendance, and that implicit measures of racial attitudes may have been impacted by the protests.

**Fig 2:**
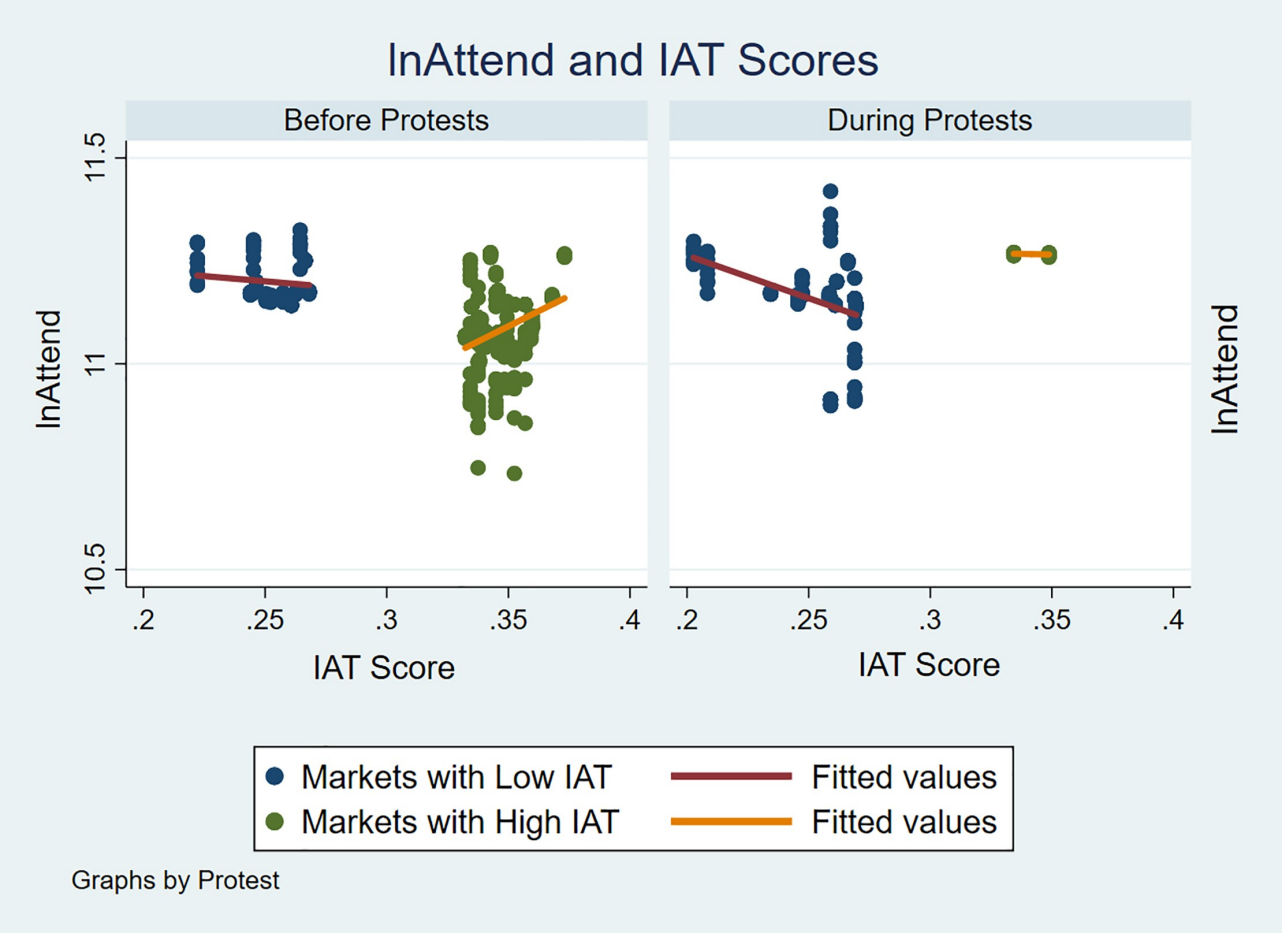
lnAttend and markets with low and high IAT scores.

Based on this, as a further robustness check to consider the fifth research question examining the effect of IAT before and during the protests, the sample used in this study was divided into markets with high and low IAT scores. From this, the attendance variables were then plotted against these variables before and after the protests to examine whether these variables had any relationship with attendance (Fig 2). In first analyzing the results for markets with low IAT scores, it was shown that before the protests there was a slight negative relationship with attendance (slope = -0.0226, *p* = 0.13). However, during the protests this relationship changed, featuring a greater downward trend with a slope of -0.0575 (*p* = 0.11) indicating that in markets with low IAT scores there was a decline in attendance at games. At the same time, it should be noted that the downward trend for low IAT scores after the protests was insignificant. Such findings match the results shown in the predictive margins from the interaction displayed in Fig 1. Next, considering the high IAT markets, there was initially an upward slope of 0.0313 (*p* = 0.08) before the protests that indicate that higher IAT scores were correlated with higher attendance at NFL games. Nevertheless, when the protests began the slope for IAT and attendance became close to horizontal with a value of -0.003 (*p* = 0.93), indicating almost no relationship between attendance and implicit bias in markets with high IAT.

In further examining the scatterplot during the protests in Fig 2, it is evident that there were only a small number of games played in markets with high IAT scores during the protests. As such, it is worth noting that the lack of games could have had an effect on the estimated results for all of the regressions, and also is likely the source of the difference for markets with high IAT in the interaction plot (Fig 1) and the robustness check (Fig 2). Overall, the scatterplots for the IAT scores show an impact from the protests, with low IAT markets moving from no relationship to a negative one, and high IAT markets moving from a positive relationship to having none. Combined with the full data for all markets, this likely could signify that the negative relationship between attendance and implicit bias from the regression models may be attributed to the markets not displayed in the scatterplot, and could emphasize that the protests had influence on consumers across markets of varying implicit racial bias.

Although the results from the estimated models and figures indicate that implicit race bias had a significant impact on attendance at NFL games, there is still need to consider the effect size for the variables in the model. In order to examine the effect size of the variables included within this study, the Eta squared values were calculated for the Models 1 and 2 and can be viewed in Table 3. Eta squared is used in this study, as prior scholars have noted its appropriateness for examining effect size in multiple regression analysis [70, 71]. In examining the values in Table 3, it is evident that all of the variables measuring racial bias, as well as their interactions with the protests are smaller than the threshold of 0.02 set out in prior research as being a small effect size in multiple regression analysis [72, 73]. Thus, while the coefficients present the argument that implicit bias may have an enduring and long-term effect on consumer demand for attendance, the effect size values indicate that any impact of implicit bias is rather inconsequential. At the same time, the effect size for the other *PewPct* and *RacialAnimus* were even smaller than the value for *IAT*, indicating that the overall influence these factors have on attendance is trivial.

**Table 3:** Effect size for base regression.

Variable	Eta Squared	DF	Eta Squared	DF
Model	0.26958	20	0.26333	16
Population	0.07869	1	0.07919	1
Per Capita Income	0.00864	1	0.00986	1
MinorityPct	0.01716	1	0.01552	1
GOPPct	0.06581	1	0.06526	1
WPCTLagHome	0.03712	1	0.03842	1
WPCTLagAway	0.00300	1	0.00304	1
Weekday	0.00239	1	0.00222	1
Holiday	0.00074	1	0.00129	1
Division	0.00005	1	0.00005	1
Relocated	0.01123	1	0.01135	1
StadiumAge	0.00527	1	0.00433	1
StadiumAgeSq	0.00615	1	0.00486	1
Protest	0.00619	1	0.00421	1
RacialAnimus	0.00004	1	0.00020	1
RacialAnimus x Protest	0.00091	1	---	---
PewPct	0.00599	1	0.00109	1
PewPct x Protest	0.00717	1	---	---
IAT	0.01570	1	0.01475	1
IAT x Protest	0.00061	1	---	---

DF = Degrees of Freedom

Shifting focus to the control variables based on prior studies examining the demand for sport, all models found that team performance as measured by lagged win percent were positive and significant for both the home and away team. These findings indicate that NFL fans were responsive to the quality of both teams in a content, and falls in line with previous studies that note the importance of the strength of both teams in determining fan interest in a sport contest [45, 63]. Next, population was insignificant in all models, while the percent of minorities living a market was positive and significant. At the same time, per capita income and the percentage of GOP voters in a market were negative and significant in most models. Overall, the demographic variable results suggest that market size and wealth may have had some impact on attendance at NFL games. Likewise, the results also suggest that markets with a lower percentage of GOP voters and higher percentage of minorities potentially had higher attendance. In this manner, *GOPPct* and *MinorityPct* not only serve as important controls within this model considering the focus on racial bias, but also highlight that demographic factors may serve as important determinants of sport demand.

Continuing to the structural factors included within the demand model, *Weekday* was positive and significant in all models, suggesting that games played on weekdays had higher attendance, likely due to the impact of Monday Night Football being of greater interest to consumers. At the same time, *Holiday* and *Division* were both insignificant in all models, indicating that games played on holidays or against division rivals did not have any impact on live attendance at NFL games. Next, the measure of teams that relocated were positive and significant in all models, indicating that teams which moved to a new market experienced an increase in attendance, likely due to the novelty effect [59]. Similarly, the results for the stadium age variable were negative and significant, indicating that fans preferred to attend games for teams that played in newer stadiums. Furthermore, stadium age squared was positive and significant in all models, indicating a concave relationship between stadium age and attendance as is commonly found in studies of sport demand [74, 56, 75].

## Discussion and conclusion

While there exists a substantial and growing body of literature examining implicit bias and racial animus [76, 48, 25, 77], recent societal events within the United States and the rest of the world highlight the importance and need for understanding the impact that the attitudes and biases towards others can have. As noted [50], small changes in implicit racial bias within a population has the capability to have large and powerful influences with ramifications for all members of society. Considering this alongside the recent events, such as the 2016 U.S. Presidential election, the emergence of white nationalism, and the Black Lives Matter protests that have all increased discussions and focus on racial issues, it suggests that we are now at a critical juncture. That is, the current setting constitutes a key point to consider how the existence of racial animus and implicit bias within the population can affect the behaviors of individuals, as well as impact organizations, governments, and other aspects of society. Based on this, the present study makes several contributes to the literature by building an economic model of attendance demand to test whether implicit bias and racial animus have an impact on consumer decisions.

From a theoretical standpoint, this study extends previous work on racial animus and explicit attitudes towards race, by examining how negative sentiments based on race can impact behavior. That is, where [48] highlighted racial animus affected the choice of voters in previous Presidential elections, we find that it did not influence the decision of consumers to attend sporting events. In considering the overall body of research using Google Trends to predict human behavior, there has been mixed findings in regards to the impact and usefulness of these types of “big data.” On one hand, Google Trends data has been utilized to show statistical relationships between racial animus and college education level [78], birth outcomes [27], and income inequality [79]. On the other hand, scholars have noted that Google Trends data is not always beneficial in terms of predicting behaviors [80], and even when there is a relationship it may only has a small effect [79]. Along these lines, the current research adds to the literature by displaying that racial animus, as measured by Google Trends data, does not necessarily predict behaviors, especially when paired with data accounting for other racial and demographic factors. Additionally, in considering the measure of racial animus developed from Google Trends in this study, it was found that the variable had a small effect size at the market level in terms of consumer interest. As such, although other studies find that Google Trends data may be able to predict behaviors within the population, it is necessary to temper these findings with the understanding of the importance these variables have.

The current research also extends previous examination of racial animus by introducing a control for implicit bias within local markets, thus showing the effect racial animus has even when accounting for other types of bias. Where previous studies find that individual choices and behaviors are often affected by measures of racial animus, we no longer find such an effect when controlling for the implicit bias in the population. Such findings differ slightly from the work of [79], who found that when including implicit bias in their model, there was only small impact of racial animus on income inequality. In this sense, it could suggest a potential connection between racial animus and implicit bias scores from individuals in the population, and thus future studies should further consider the relationship that may exist between racial animus and implicit bias. As such, the current study helps to expand the understanding of the behaviors implicit bias, especially in regards to how such bias can affect larger populations and organizations. That is, where implicit bias research often focuses on the psychology of individuals and examining factors that help to predict the formation of such attitudes, this study emphasizes how implicit bias may affect larger patterns of consumer behavior. In turn, any negative effect of implicit bias on sport consumption not only is a source of inefficiency in the market, but also can have negative consequences for sport organizations and related stakeholders. Along these lines, if implicit bias is able to affect the behavioral patterns of large groups of consumers, the presence of bias could thus present dangers for society at a number of levels. However, in considering the effect size for implicit bias, it indicates that any impact on attendance from this measure was likely negligible, and thus may not have as wide reaching implications as previous research has theorized.

Another important contribution of this research is in extending the understanding of activist movements, and the societal response to them. Within management and economic sociology, researchers have argued that social movements, such as protests, have the ability to influence the resources of organizations, and thus cause disruption in the marketplace [81, 82, 83]. Overall, the results from the models in this research indicate that it was not just the presence of the protests which influenced attendance at NFL games, but rather the level of implicit bias in markets that experienced the protests. As such, these findings extend the previous understanding of social movements in the management literature, which often conclude that the presence of protests are the source of market disruption. Rather, this study notes that the interaction of the protests with other socio-economics factors may be what leads to disruptions in the marketplace. At the same time, the effect size for the protest variable and its interactions with the various measures of racial bias in this study were quite small. As such, it may suggest that while previous studies have found significant changes in market behaviors based on protests, the actual effect these protests had may be somewhat inconsequential. Although researchers have widely examined the impact of market disruption, few have studied how market level factors could influence the effectiveness of movements. Thus, the findings from this research provide an empirical advancement by incorporating market level measures to analyze how consumers respond to protests.

Next, the current research also contributes to the economics and sport literature, in emphasizing the importance of implicit bias and racial animus, especially in relation to athlete activism. There exists significant lineage of research in sport analyzing biases and discrimination within society [84, 85, 86], and the influence it has on individuals and organizations [87, 88, 89]. However, implicit bias and racial animus have not been widely considered within these studies, likely due to a lack of awareness of the issue of racial animosity and inability to operationalize the concept empirically. In this manner, the current research represents both a theoretical and empirical contribution through its discussion and inclusion of the concept of racial animus. Furthermore, though sports economics research has analyzed how consumer decision to attend games can be influenced by the racial composition of teams [90], such research has ignored the racial attitudes and demographics of the markets. Thus, through including data to measure racial animus, implicit race bias, and racial/political demographics within a demand model, this study advances the empirical examinations of the impact of race on sport consumption. In further discussing the empirical significance of this study, it is noted that the use of big data to aggregate human behaviors is becoming more prevalent, especially in trying to understand complex human behavior. However, Google Trends remains an underutilized tool in conducting these type of analyses, and thus signifies another contribution of this study.

Continuing, the current study also has important implications for managers, especially those who work in sport business. Generally, the results from this research indicate that implicit race bias has the ability to influence the consumer decision-making process, but that the size of this effect is minor. Nevertheless, racial implicit bias is a behavior that organizations, managers, and other stakeholders need to be cognizant of, as they could influence the operations and accrual of resources. Moreover, the findings also emphasize the impact that higher levels of negative racial sentiment in the population can have on the choices of individuals. That is, it suggests that in the current environment where white nationalism and other forms of racial animus are evident within society, there are economic and social consequences that can affect the market. This is certainly the case in the sport industry, as recent news reported the growing presence of far-right members in the supporter groups of New York City Football Club (NYCFC), some of whom have taken part violent acts [91]. Although many fan groups have tried to push back against the growth of fascist attitudes and symbols in the stands [92], league commissioner Don Garber stated that it is not the job of the league to “judge and profile any fan… The last thing we’re going to do is start getting into profiling people and what their backgrounds are” [93]. While such approach to not alienating any specific fan group is often utilized by businesses seeking to maximize profits, the findings from this research indicate that passive strategies of avoidance can still lead to a decline in consumer interest, and thus is not a solution to dealing with extremism and negative racial sentiments.

In considering the potential limitations of this study, it needs to be acknowledged that there are many ways in which racial animus and implicit bias can be measured. As such, the measures utilized within this study do not encompass all types of behaviors that are representative of racial animus or implicit racial bias. However, for each of these constructs we included an individual variable to account for the different types of explicit racial attitudes and implicit racial bias. Certainly, future studies should seek to expand on this current research by utilizing other measures of racial animus and implicit bias, as well as testing to see if such behaviors affect organizations outside of sport. The next limitation in this study is that the racial composition of NFL teams was not accounted for. Previous studies of sport demand have noted bias and discrimination from consumers in baseball and basketball [90, 94], suggesting that the racial composition of teams could influence fan interest. However, controlling for such factors in the NFL is rather cumbersome, as rosters are composed of over 60 players which shift weekly, compared to around a dozen for professional basketball. As such, where the presence of a single player of a certain race can affect demand in basketball [90], it is less likely to have an impact in the NFL because of the number of players on a team and their relative lack of visibility. The final limitation in this research is in considering whether the protests had an impact on attendance at NFL games, or whether other events could have had an impact on attendance. Although 2016 was an eventful year that included a combative Presidential election, increased tensions in domestic and international politics, etc., it is worth noting that the NFL protests intersected with many of these events. As such, though the protests may not be the only important event of 2016, their connection with media and the contentious politics of the Presidential election would suggest they certainly played an integral role in impacting attendance. In concluding, based on the findings in this study, there is need for future research to continue advancing examinations of the influence that racial animus and implicit racial bias have within society. Such studies would not only have wide-ranging impact within various disciplines in academia, but also would build upon the critical understanding of the changing socio-cultural dynamics presented in this research.
